# Profibrotic Signaling and HCC Risk during Chronic Viral Hepatitis: Biomarker Development

**DOI:** 10.3390/jcm10050977

**Published:** 2021-03-02

**Authors:** Alessia Virzì, Victor Gonzalez-Motos, Simona Tripon, Thomas F. Baumert, Joachim Lupberger

**Affiliations:** 1 Université de Strasbourg, 67000 Strasbourg, France; virzi@unistra.fr (A.V.); gonzalezmotos@unistra.fr (V.G.-M.); simona.tripon@chru-strasbourg.fr (S.T.); thomas.baumert@unistra.fr (T.F.B.); 2 Institut National de la Santé et de la Recherche Médicale, U1110, Institut de Recherche sur les Maladies Virales et Hépatiques (IVH), 67000 Strasbourg, France; 3 Institut Hospitalo-Universitaire, Pôle Hépato-Digestif, Nouvel Hôpital Civil, 67091 Strasbourg, France; 4 Institut Universitaire de France (IUF), 75231 Paris, France

**Keywords:** HBV, HCV, biomarkers, liver disease, HCC, cure, risk

## Abstract

Despite breakthroughs in antiviral therapies, chronic viral hepatitis B and C are still the major causes of liver fibrosis and hepatocellular carcinoma (HCC). Importantly, even in patients with controlled infection or viral cure, the cancer risk cannot be fully eliminated, highlighting a persisting oncogenic pressure imposed by epigenetic imprinting and advanced liver disease. Reliable and minimally invasive biomarkers for early fibrosis and for residual HCC risk in HCV-cured patients are urgently needed. Chronic infection with HBV and/or HCV dysregulates oncogenic and profibrogenic signaling within the host, also displayed in the secretion of soluble factors to the blood. The study of virus-dysregulated signaling pathways may, therefore, contribute to the identification of reliable minimally invasive biomarkers for the detection of patients at early-stage liver disease potentially complementing existing noninvasive methods in clinics. With a focus on virus-induced signaling events, this review provides an overview of candidate blood biomarkers for liver disease and HCC risk associated with chronic viral hepatitis and epigenetic viral footprints.

## 1. Introduction

Chronic liver disease is a major health problem and globally associated with > 2 million deaths per year [1]. The most important etiologies are chronic viral hepatitis, alcohol abuse and metabolic dysfunction-associated fatty liver disease (MAFLD) [2], sharing a similar pattern of liver disease progression from chronic inflammation, fibrosis to terminal complications, such as decompensated liver cirrhosis and liver cancer [3,4,5,6]. Globally, every fourth cancer-associated death is associated with liver cancer, most frequently hepatocellular carcinoma (HCC), with a fast-rising incidence [7]. HCC typically arises in the background of cirrhosis; however, in HCV patients, about 10% of cases can develop in a noncirrhotic liver [8].

Despite tremendous advances in antiviral therapies, chronic viral hepatitis B and C are still the major etiology for chronic liver disease. Worldwide, an estimated 180 million people live with hepatitis B virus (HBV) and 75 million with hepatitis C virus (HCV), and for most, testing and treatment remain beyond reach [9]. Both viruses share similar as well as distinct mechanisms contributing to liver disease and cancer. In Europe, it is estimated that 10–15% of HCCs are caused by HBV infection, while 70% are caused by HCV infection, HCV being the major risk factor for HCC development [10]. Both viruses contribute to liver fibrosis and HCC risk by multiple factors involving a dysregulation of host signal transduction through viral proteins, miRNAs, virus-induced growth factor and cytokine expression or antiviral responses that cumulate in a pro-fibrotic and pro-oncogenic environment in the liver [11,12,13,14,15,16,17]. Liver fibrosis is characterized by an excessive production of the extracellular matrix by hepatic stellate cells and myofibroblasts in response to the inflammation and oxidative stress induced by viral infection [18].

The most important measure to reduce HCC risk is to eliminate the underlying etiology. However, even though viral infection with HBV or HCV can be controlled or cured, the risk of developing HCC cannot be fully eradicated due to multiple reasons, especially in patients with already advanced liver disease. The mechanisms are not well understood, but evidence points towards epigenetic viral footprints that maintain dysregulated pro-oncogenic signal transduction. It is thus crucial to be able to identify patients with elevated HCC risk to stratify for a more frequent liver screening. Today, liver disease diagnosis and HCC risk assessment relies on a combination of imaging, blood markers and liver biopsies. While liver tissue allows a detailed transcriptomic HCC risk assessment linked to predictive transcriptomic signature [19,20], liver biopsies are associated with a significant risk for the patients and, therefore, are not applicable for a tighter screening [21]. An impressive number of approaches and “liquid biomarker” candidates for liver disease are underway, studying extracellular vesicles, circulating tumor cells and cell-free nucleic acids (reviewed in detail [22]) to improve prognostic power, minimize the risk for the patients and provide additional tools for the screening of patients at risk. With a focus on virus-induced signaling events, this review provides an overview of the candidate blood biomarkers of fibrotic liver disease and HCC risk associated with chronic viral hepatitis.

## 2. Viral Hepatitis B and C

Although HBV and HCV are hepatotropic, causing similar liver disease, they are very different viruses. HBV is an hepatotropic DNA virus of the Hepadnaviridae family, which specifically infects hepatocytes via the recently discovered functional receptor sodium taurocholate co-transporting polypeptide (NTCP) [23] and epidermal growth factor signaling [24]. Following endocytosis, the nucleocapsid is released into the cytoplasm, and the partially double-stranded viral relaxed circular DNA (rcDNA) is repaired and converted to covalently closed circular DNA (cccDNA) in the nucleus (for a more detailed review, see [25]). cccDNA is a replicative intermediate of the HBV life cycle, and it is crucial for HBV persistence within the hepatocytes. It serves as a template for the transcription of HBV RNA species and consequently for the translation of HBV proteins, i.e., three surface proteins (L-HBsAg, M-HBsAg and S-HBsAg), core (HBc), E antigen (HBeAg), X protein (HBx) and viral polymerase (Pol). Among them, HBx is believed to have key roles related to HBV replication and signaling pathways [26].

HCV is a single-stranded, positive-sense RNA virus of the Flaviviridae family that requires multiple host entry factors, including receptor tyrosine kinase signaling. After endocytosis, the RNA genome is translated into a viral polyprotein at the endoplasmic reticulum, leading to a massive reshaping of host membranes to a replication complex termed the membranous web. De novo virus assembles at lipid droplets, which are accumulated at the replication complex (for a more detailed review, see [27,28]). HCV does not integrate into the host genome nor possesses a latent viral phase. Thus, HCV requires a constant modulation of the host cell to evade the antiviral response and to maintain its viral cycle [29,30,31]. In contrast to HBV, which is considered to be a stealth virus that does not cause big changes in the host transcriptomics [32], HCV massively re-orchestrates signaling pathways. A multiomics analysis of HCV infection studying gene set enrichment analysis (GSEA) revealed that almost half of the ~2000 studied gene sets from the molecular signature database (MSigDB) were dysregulated by HCV infection involving pro-oncogenic pathways regulating proliferation (EGF/MAPK), inflammation and stress (STAT3, NF-κB), hypoxia and angiogenesis (VEGFR) and fibrosis (TGF-β) [16,17]. Such massive remodulation of the signaling landscape holds the potential to derive novel minimally invasive biomarkers.

## 3. Antiviral Therapies

The most important measure to treat liver disease and prevent HCC formation is the removal of the underlying etiology. The challenges to help patients with viral hepatitis are heterogenous: while an efficient preventive HBV vaccine is available, established chronic HBV infection can only be controlled but rarely eliminated due to a persistent chromosome-like viral DNA species and genome integrations [33]. A “functional cure” of HBV infection is defined by a sustained loss of hepatitis B surface antigen (HBsAg) in the blood, with or without seroconversion to anti-HBsAg. However, it is not always achieved in individuals with HBV. For this reason, the achievement of a sustained HBsAg seroclearance, even after suspension of the antiviral therapy, is nowadays considered the most realistic endpoint for the cure of individuals with chronic HBV. On the other hand, “virological cure”, defined as the complete eradication of the virus, is too hard to reach, and it does not represent a reasonable therapeutic goal to date. This is due to the integration of HBV DNA into the host genome and the persistence of cccDNA within the hepatocytes [34]. Importantly, a constant activation state of fibrotic signaling pathways is believed to persist even in patients with undetectable HBV serum viral loads after therapy [35,36,37].

For HCV, no vaccine is in reach, but the novel generation of antiviral therapies with direct acting antivirals (DAAs) can efficiently cure HCV infection [38]. A new generation of interferon-free HCV drug regimens (sofosbuvir/velpatasvir and glecaprevir/pibrentasvir) is pan-genotypic and, therefore, can be used to treat individuals without identifying their HCV genotype and subtype [38]. Efficiency is very high (90–98% after 12 weeks; ASTRAL-3 trial) even in patients with difficult to treat genotype 3 and advanced liver disease [38]. However, the high price for HCV cure is still a barrier to guarantee treatment programs worldwide. Even when prices vary across countries, there is no adjustment of DAA prices with population income or viral infected population, making its accessibility more complicated for the poorest sectors of society [39]. HCV cure markedly decreases but cannot fully eliminate HCC risk, especially in patients with already advanced liver disease [40].

## 4. Fibrosis and HCC Screening in the Clinics—State of the Art

The majority of HCCs arise from liver cirrhosis, and thus the current HCC risk assessment is largely coupled to the staging of liver fibrosis. Despite the screening programs in cirrhotic patients, often the HCCs are diagnosed at an advanced stage. Less than 30–40% of them are eligible for a curative treatment using surgical approaches or radiological ablation [41]. Currently, the trans-abdominal ultrasound surveillance of patients at risk is the standard technique to detect HCC. However, the sensitivity of this method is not good enough to detect small tumors. For this reason, other methods, e.g., computed tomography (CT) or magnetic resonance imaging (MRI), are added, increasing cost, complexity and time for early detection and diagnosis. Magnetic resonance elastography (MRE) can be thought of as quantitative, noninvasive palpation. The use of this technique has become widespread in the diagnosis and staging of liver fibrosis [42]. However, early detection of HCC is complicated because of the co-existence with a chronic liver disease. The performance of noninvasive methods is represented by the area under the receiver operator characteristic (AUROC) curve, which provides information regarding the sensitivity and specificity of the method. Based on this, several tests have been proposed, such as the fibrosis-4 index or fibrotest, which may help clinicians in determining prognosis and risk for future complications [43]. Another noninvasive method that helps in discriminating cirrhotic patients from noncirrhotic patients is the measurement of liver stiffness using transient elastography (TE), as the fibrotic tissue is much stiffer than healthy tissue. In patients with advanced fibrosis, the liver function and patient prognosis is classified with the Child–Pugh score summarizing biological and clinical features, i.e., bilirubin, albumin, prothrombin time, ascites and hepatic encephalopathy. Child–Pugh comprises 3 classes of severity: A, good liver functionality (median 2-year survival: 85%); B, moderate liver functionality (median 2-year survival: 57%); C, poor liver functionality (median 2-year survival: 35%) [44].

Several patient-derived transcriptomic signatures that associate with HCC risk and which are specific for certain etiologies or reflect a hepatic state of struggle in the liver independently from the underlying etiology have been identified in recent years (for a more detailed review, see [45]). Although a translation into minimally invasive biomarkers is explored, these signatures largely depend on liver tissue from resections or liver biopsies, which are still the gold standard to diagnose liver disease and assess HCC risk. However, liver biopsies are costly, exhausting for the patients and associated with a significant risk, reasons why this procedure is not applicable for a tighter screening [21].

Current guidelines recommend the screening of HCC in at-risk patients using ultrasonography (US) of the liver every 6 months with or without serum alpha-fetoprotein (AFP) [46,47], the most commonly used biomarker for liver disease detection. Additional conventional serum biomarkers are lectin-binding AFP-3 (AFP-L3) and des-carboxyprothrombin (DCP), which are still evaluated [48,49,50,51,52]. A Japanese prospective study demonstrated that a combination of DCP with AFP level is useful to detect HCC development and recurrence in chronic liver disease patients [53]. This was recently consolidated by a retrospective study, which showed that combining DCP and AFP serum levels in NUC-treated HBV Caucasian cirrhotic individuals, represents a potential surveillance strategy for HCC [49]. Additional candidate biomarkers for HCC in the blood have been suggested, i.e., proprotein convertase subtilisin/kexin type 9 (PCSK9) [54,55], glypican 3 (GPC3), squamous cell carcinoma antigen (SCCA), cytokeratine-19, osteopontin (OPN), Golgi protein-73 (GP73), alpha-L-fucosidase (AFU) [56], heat shock 70 kD protein (HSP-70) [57], annexin A2, midkine (MDK), aldo-keto reductase family 1 member B10 (AKR1B10) [58], and HCC-responsive miRNAs and cell-free DNA (for a more detailed review, see [59]). However, even if these candidate biomarkers are promising, to date, none of them have been adopted in the current clinical practice, and they need to be externally validated.

Additional scoring systems have been predictive for HCC risk in patients with chronic liver disease. The GALAD score [60,61] is derived from the combination of different parameters and single biomarkers, such as gender, age, AFP-L3, AFP and DCP, and has been validated in several patient cohorts [62]. The ALBI score evaluates the liver function of patients with HCC of different stages based on albumin and bilirubin levels in the blood [63]. However, the complex mathematical calculation of the ALBI score has limited its use, and new scores based on it have been developed, e.g., EZ-ALBI [64] or the modified ALBI (mALBI) which is used in clinical practice [65]. The enhanced liver fibrosis (ELF) score assesses a range of liver disease in conjunction with liver biopsy [66]. It provides a single score combining in an algorithm the measurement of three indirect biomarkers: HA, PIIINP and TIMP-1. The algorithm detects accurately liver fibrosis in patients with chronic HCV [67]; however, it is limited in low disease prevalence [68].

## 5. Signaling Pathways Associated with Candidate Serum Biomarkers

**Figure 1:**
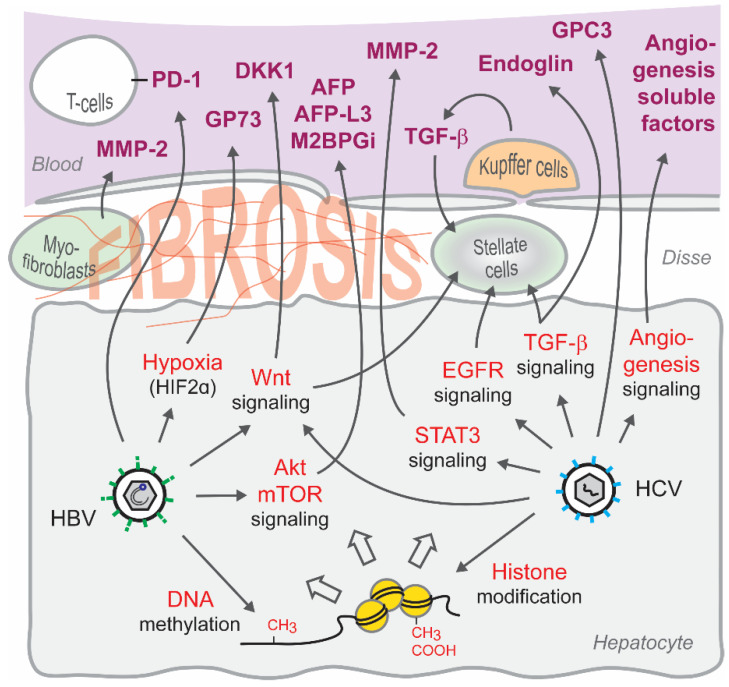
Dysregulation of signaling pathways by chronic viral hepatitis and epigenetic imprinting impact the secretion of circulating candidate biomarkers to the blood. Abbreviations: AFP, alpha-fetoprotein; AFP-L3, Lectin-Binding AFP-3; Akt, AKT Serine/Threonine Kinase; DKK1, Dickkopf WNT Signaling Pathway Inhibitor 1; Disse, Space of Disse; EGFR, Epidermal Growth Factor Receptor; GPC3, Glypican 3; GP73, Golgi Membrane Protein 1; HIF2α, Hypoxia-Inducible Factor 2 Alpha; M2BPGi, mac-2-binding protein glycan isomer; MMP-2, Matrix Metallopeptidase 2; mTOR, Mechanistic Target Of Rapamycin Kinase; PD-1, Programmed Cell Death 1; STAT3, Signal Transducer and Activator of Transcription 3; TGF-β, Transforming Growth Factor Beta; Wnt, Wnt Family Member.

Signal transduction is an essential process involved in almost every step of cellular homeostasis. Signaling is tightly controlled, transmitting signals between cellular compartments and regulating gene transcriptional responses. Thus, the chronic dysregulation of signaling pathways is involved in the majority of diseases, including cancer [69]. Viruses including HBV and HCV make use of host signaling to maintain their life cycles or to evade the host antiviral response. The resulting persistent dysregulation of host signaling pathways by chronic viral infection promotes viral pathogenesis and malignant transformation [70]. Therefore, the study of virus-dysregulated signaling pathways may contribute to identify efficient minimally invasive biomarkers for liver disease (Figure 1).

Despite differences in terms of structure and life cycle, HBV and HCV are believed to share common pathways which influence hepatic fibrosis and promote hepatocarcinogenesis [71,72]. It became evident that HBV infection does not trigger the innate immune response and thus behaves as a stealth virus in the liver [73,74]. Nevertheless, chronic HBV infection impacts host signaling with potential relevance to markers of liver disease progression. The HCC biomarker AFP is also a regulator of growth signaling via PI3K/AKT signaling in hepatoma cells [75]. The viral protein HBx induces the expression of AFP, potentially driving the malignant transformation of hepatocytes in vivo via activation of the PI3K/AKT/mTOR pathway [76,77]. Moreover, members of the mTOR signaling pathway and eukaryotic translation initiation factors (eIFs) have recently been identified as potential biomarkers for HCC, and their expression patterns depend on different HCC aetiologias, such as HBV, HCV and non-virus-related HCC [78]. Another potential HBV-responsive risk marker is the protein Dickkopf WNT Signaling Pathway Inhibitor 1 (DKK1) involved in embryonic development as an inhibitor of Wnt signaling. DKK1 is a secreted protein whose mechanism of action is centered in binding and isolating the low-density lipoprotein receptor-related protein 6 (LRP6) co-receptor avoiding its role in activating the Wnt signaling pathway. DKK1 promotes HCC development by the modulation of the Wnt/β-catenin signaling pathway [79] and interaction with TGF-β signaling [80,81]. High serum levels of DKK1 may distinguish HCC associated with chronic HBV infection from HCC associated with nonviral liver cirrhosis. Moreover, DKK1 may allow early-stage HCC detection even in patients with AFP negative status [82]. DKK1 is also gaining interest as a potential biomarker for HCV-associated HCC. Although HCV core protein promotes the activation of the Wnt signaling protein and the suppression of Wnt pathway inhibitors [83,84], DKK1 abundance seems to be significantly decreased in the blood of patients with HCV [85]. However, DKK1 is spiking in patients with HCV who also have HCC [86].

GP73 has been suggested as a serum biomarker for liver cirrhosis in individuals with chronic HBV [87]. Moreover, GP73 seems to be a good predictor of liver inflammation and fibrosis in HBV patients with normal or slightly raised alanine aminotransferase (ALT) [88]. The biological function of GP73 is not completely understood but it is assumed to be involved in protein secretion and signaling. Moreover, its expression is linked to different pathological conditions [89]. HBV modulates various signaling pathways converging in GP73 modulation [90,91]. For example, it promotes GP73 expression by the activation of hypoxia-inducible factor-2α (HIF-2α) signaling [91], which is a hallmark of chronic infection and HCC development. Moreover, GP73 influences the immune response to HBV infection, as an increased production of GP73 can be observed in HBV-stimulated leukocytes [90], in peripheral blood mononuclear cells isolated from healthy donors and in macrophages derived from human acute monocytic leukemia cells (THP-1). In the same study using hepatoma cell lines, the authors demonstrated that GP73 represses the expression of the p50 subunit of NF-κB, promoting HBV replication and thus highlighting the role of GP73 as a potential antiviral modulator [90]. Immune dysregulation and T-cell exhaustion are among the major hallmarks of chronic HBV infection and the associated pathological development [92]. On this basis, the presence of HBV-specific T cells has been recently proposed as an immunological biomarker for safely monitoring therapy in chronic HBV patients [93] and programmed cell death protein (PD-1) expression as a potential marker for liver fibrosis in patients with chronic HBV [93].

Recently, mac-2-binding protein glycan isomer (M2BPGi) has been shown to enhance the aggressiveness of HCC via the activation of the mTOR signaling pathway [94], harboring potential as a minimally invasive biomarker. Glycoproteomic analysis has revealed that mac-2-binding protein (M2BP), an extracellular matrix protein that interacts with collagens, fibronectin and integrin [95], may undergo specific changes in its glycan structure correlating with fibrosis development [95,96]. M2BPGi has been suggested as a valid predictor of fibrosis and HCC in HBV patients [97,98,99,100]. Moreover, according to a prospective study conducted in China, M2BPGi serum level decreases in chronic HBV patients treated with nucleos(t)ide analogues (NAs), indicating its potential role in predicting HCC development in NA-treated populations [101]. In addition, M2BP-modified molecules have been studied as biomarkers of fibrosis in patients with chronic HCV infection [102]. Its cut-off values differ between etiologies, and M2BP levels decrease after viral cure [103]. Interestingly, M2BPGi has been introduced as a novel and noninvasive biomarker for the assessment of liver fibrosis in chronic HCV patients treated with DAAs [104].

Signaling pathways play a pivotal role during viral hepatitis and liver fibrosis. Epidermal growth factor receptor (EGFR) signaling is required by HBV and HCV for entry [24,105], where it orchestrates entry factor complex formation and endocytosis [24,106]. Importantly, HCV itself promotes EGFR signaling [12,16,107] to maintain its life cycle and to attenuate the host antiviral response [106] with important consequences for liver disease progression. Indeed, EGFR signaling has been identified as a major driver of liver fibrosis and HCC in animal models and patients [13,19]. Additionally, TGF-β signaling is induced by HCV infection in hepatocytes [16] and in activated Kupffer cells, which are resident liver macrophages activated during liver injury [108]. Like EGF and Wnt signaling, TGF-β is a major regulator of cell proliferation, differentiation and apoptosis. It is essential for the induction of epithelial–mesenchymal transition (EMT) and the activation of stellate cells [108]. TGF-β is a cytokine suppressing tumor activity at early stages by arresting cell growth and inducing apoptosis. However, at later tumor stages, it promotes the proliferation and survival of malignant cells (for a more detailed review, see [109]). HCV infection induces TGF-β signaling indirectly via NF-kB and unfolded protein response (UPR) [110] and directly via the interaction of HCV core protein with SMAD3 [111]. HCV core protein increases intrahepatic and circulating levels of endoglin, which is a TGF-β1 co-receptor associated with progressive hepatic fibrosis during chronic HCV infection [112].

Persistent oxidative stress is an important factor in virus-induced liver fibrosis. Especially HCV infection, and its massive reorganization of cellular membranes to the replication complex, is a major cause of UPR and oxidative stress [113]. HCV protein core, NS3 and NS5, block heme oxygenase-1 (HO-1) in hepatocytes accumulating oxygen radicals in the cell [114]. This activates NF-kB and STAT3 [113], which are key players in inflammation and cancer [115]. During HCV infection, STAT3 activity is further intensified by a suppression of negative regulators, i.e., the STAT3 phosphatase PTPRD via miR135a-5p [17] and SOCS3 by enhancing EGFR signaling [106]. Consequently, STAT3 signaling impairs peroxisomal function, leading to an accumulation of very-long-chain fatty acids and peroxides in the HCV-infected hepatocyte [16]. Moreover, HCV-induced STAT3 signaling also triggers the upregulation and secretion of the metalloprotease MMP-2 [116], which is involved in remodeling the extracellular matrix and has been previously suggested as a prognostic marker for liver fibrosis [117].

HCV infection induces hypoxia in infected cells and stabilizes HIFs [118], which is also a hallmark of HCC development linked to a stimulation of angiogenesis. In patients with HCV-associated cirrhosis and HCC, several angiogenesis soluble factors were significantly upregulated in the blood plasma, including TIMP-1, TIMP-2, HGF, angiopoietin 1, angiopoietin 2, VEGFA, IP-10, PDGF, KGF and FGF. AUROC analysis highlighted especially the potential of angiopoietin 2, a growth factor that belongs to the angiopoietin/Tie signaling pathway [119,120,121]. Additionally, CCL20, a secreted chemokine detected in HCCs, promotes blood vessel formation during chronic HCV infection [122]. CCL20 and VEGF correlate in patients with cirrhosis and HCV-induced HCC, highlighting their potential as biomarkers for HCV-induced HCC [123,124,125]. Moreover, the serum level of TIMP-1 has been found to be significantly correlated with fibrosis development in chronic HBV patients [126]. Interestingly, a previous study demonstrated that TIMP-1 and hyaluronic acid (HA) are good predictors of advanced liver fibrosis in chronic hepatitis B and D patients [127] and bases for the earlier mentioned ELF score.

Glypican-3 (GPC3) is an heparan sulfate proteoglycan that regulates cell morphology via the Hippo/YAP pathway. In a normal liver, the HCV entry factor CD81 interacts with GPC3 and inhibits the Hippo/YAP pathway. HCV E2 protein mimics the role of CD81 stimulating Hippo/YAP by engaging GPC3. In a chronically inflamed liver, HCV is thus likely to promote hepatic neoplasia by the growth of early CD81-negative neoplastic hepatocytes, which are resistant to HCV infection [128]. Given its upregulation in the blood of patients with HCV-associated HCC, GPC3 had been suggested as a biomarker [129].

## 6. Virus-Induced Epigenetic Changes as Biomarkers

Epigenetic imprinting acts as a memory for environmental influences and disease [130]. This has long-term consequences to the cellular homeostasis and pathogenesis relevant not only for therapeutic strategies but which may also be instrumental to identify specific biomarkers. Epigenetic modifications comprise DNA methylation and posttranslational modification of histones, which directly regulates the accessibility of genes to the transcriptional machinery but also posttranscriptional regulation via noncoding RNAs [131]. The discovery of new epigenetic modulators is paving the way to the identification of new epigenetic biomarkers for the development of diagnostic and prognostic tools for hepatic fibrosis. It has been demonstrated that aberrant epigenetic signatures associated with fibrosis and HCC are released into the blood stream, providing blood-based biomarkers that could be used for advancing the diagnosis and prognosis of liver-associated diseases [132,133]. During chronic viral hepatitis, specific DNA methylation patterns in the genes of peripheral blood mononuclear cells (PBMCs) suggest a role in the progression of liver disease to HCC [134]. Methylation of cytosine-phospho-guanine (CpG) dinucleotides island in regulatory gene elements correlates with the silencing of the gene expression. Thus, a methylome analysis can identify dysregulated disease-relevant signaling pathways. This strategy highlighted a role of dysregulated IL-15, IL-8, as well as nitric oxide signaling in PBMCs from HBV patients and cirrhotic livers causing reprogramming of the immune and inflammatory responses [135]. HBV causes a virus-specific DNA methylation pattern in the hepatocyte DNA [136], which, however, in a study from 2015 on primary human hepatocytes had only limited overlap with the transcriptional pattern [137]. Circulating methylated DNA fragments are explored as a noninvasive diagnostic tool for early-stage liver cancer prevention [138,139,140]. Interestingly, this also includes methylated fragments of the SOCS3 gene [139], which is a negative regulator of the IL-6/STAT3 signaling pathway.

Epigenetic regulation of gene expression by HCV has been observed at the histone level. Such epigenetic footprints have been identified in cell lines and patients with chronic HCV infection and NASH [141,142,143], suggesting a lasting dysregulation of signaling pathways even after the underlying cause has eased. Indeed, comparative ChIP-seq and RNA-seq analysis of DAA-cured HCV patients identified epigenetic histone modifications. These were associated with the dysregulated pro-oncogenic transcriptional pattern, suggesting a persistently dysregulated signal transduction after viral cure [141]. Comparative analysis with HCV-infected and DAA-cured human liver chimeric mice highlighted an HCV-specific viral footprint, since these mice do not develop liver fibrosis, which also involves the STAT3 phosphatase PTPRD [141]. Moreover, a liver fibrosis-specific footprint of cancer-risk genes has been identified in HCV- and NASH-associated fibrotic patients’ livers [142]. However, a translation of these footprint signatures into blood born biomarkers is pending.

HCV infection and liver disease largely impact miRNA expression [11], which influences signaling pathway activity and liver disease progression [17]. Circulating miRNAs harbor the potential of being developed into minimally invasive biomarkers [144,145]. miRNAs are enriched and well protected in extracellular vesicles (EVs) in the blood. Exosomal miRNAs have been evaluated in clinics, such as miR-122 and miR-21, for the early detection and prediction of HCC [146], and let-7s for the detection of liver fibrosis in patients with chronic hepatitis C infection [145,147].

## 7. Discussion and Perspectives

Although HBV and HCV cause both liver disease progression and HCC, the clinical challenges differ substantially. HBV infection can only be controlled but not eradicated because of a persisting chromosome-like cccDNA and genome integration [36]. HCV infection is now curable; however, chronic infection is leaving an epigenetic footprint that manifests the dysregulation of the pro-oncogenic signal beyond viral eradication. In both cases, patients remain at considerable risk to develop HCC over the years, which highlights the importance of reliable and minimally invasive biomarkers to stratify these risk patients for tighter HCC screening. A combination of circulating signaling components, secondary markers (e.g., gut microbiome [148,149]), with noninvasive imaging biomarkers will hold the biggest potential. However, it is important to remark that although some of these signaling-linked candidate biomarkers show promising results, almost all of them are still in development, and only AFP has reached phase V (Table 1). Moreover, it is generally difficult to draw a line between biomarkers derived from the pro-fibrotic signaling events and carcinogenesis markers during end stage liver disease. While the dysregulated signaling and epigenetics in diseased livers partially overlap between etiologies, e.g., HCV and MAFLD [13,16,141,142,150], hepatocarcinogenesis is a highly heterogenous event even within a specific etiology [151,152]. This, we need novel concepts, which differentiate those markers that “simply” predict increased fibrogenesis from those that are definitively associated with risk of carcinogenesis.

While HCV infection was rendered a curable disease due to efficient direct antiviral therapies, an important unmet medical need is to identify the fraction of patients with elevated HCC risk. In future, etiology-specific epigenetic markers, including histone modifications and miRNAs, will provide new perspectives for translation into correlating secreted biomarkers, which may be used for personalized approaches targeting specific groups of patients, e.g., biomarkers for HCC risk in HCV-cured patients.

## Figures and Tables

**Table 1:** Promising minimally invasive biomarker candidates with links to virus-induced signaling (HBV, HCV) and predictive of liver fibrosis and HCC. Biomarker research is categorized into phases I–V [153]: phase V (evaluates the effect of the biomarker screening in the burden of the disease in the population), phase IV (prospective evaluation of the biomarker to assess its clinical performance), phase III (testing in patients before their diagnosis to determine the performance of the biomarker in detecting pre-clinical disease), phase II (testing in patients at high risk to determine the performance of the biomarker in distinguishing between patients with and without the disease), phase I (discovery of new biomarkers by investigating gene expression and protein levels in pathological tissue and patient samples).

Biomarker	Specificity	Viral Etiology	Development	Reference
			Status	
AFP	HCC	HBV, HCV	Phase V	[41,46,47,51,52,77,101]
DCP	HCC	HBV, HCV	Phase IV	[48,49,50,51,52,53]
M2BPGi	HCC	HBV, HCV	Phase IV	[95,96,97,98,99,100,101,102,103,104]
MDK	HCC	HCV	Phase III	[81,154]
OPN	HCC, fibrosis	HBV, HCV	Phase III	[155,156,157]
Annexin A2	HCC, fibrosis	HBV, HCV	Phase II	[158,159]
DKK1	HCC	HBV, HCV	Phase II	[81,82,85,86]
GPC3	HCC	HCV	Phase II	[128,129]
HSP-70	HCC	HBV, HCV	Phase II	[57]
PCSK9	HCC	HCV	Phase II	[54,55]
SCCA	HCC, fibrosis	HCV	Phase II	[160,161]
TIMP-1	Fibrosis	HBV, HCV	Phase II	[119,126]
Angiopoietin-2	HCC, fibrosis	HCV	Phase I	[119,120,121]
CCL20	HCC	HCV	Phase I	[122,123]
Endoglin	Fibrosis	HCV	Phase I	[112]
VEGF	HCC, fibrosis	HCV	Phase I	[119,123,124,125]
